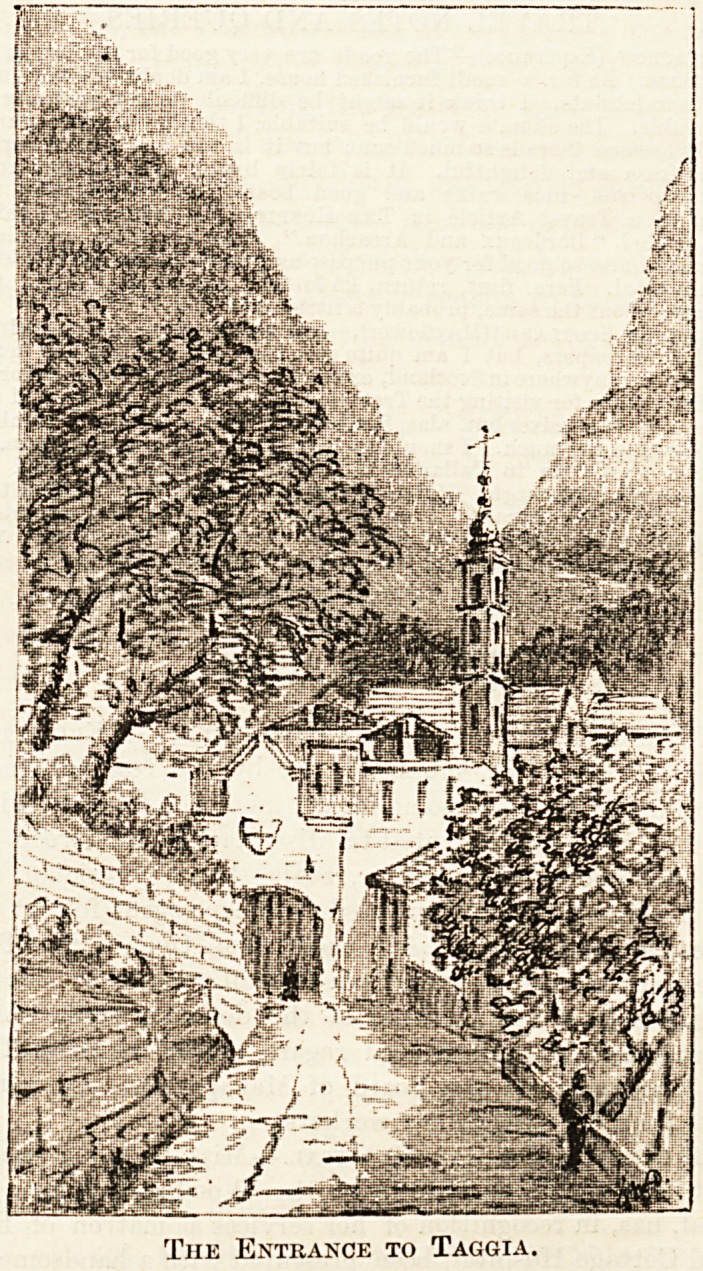# "The Hospital" Nursing Mirror

**Published:** 1899-07-01

**Authors:** 


"The Hospital" Nursing Mirror.

Being the Nursing Section of "The Hospital."

[Contributions for this Section of "The Hospital" should be addressed to the Editor, The Hospital, 28 & 29, Southampton Street, Strand, London, W.C., and should have the word "Nursing" plainly written in left-hand top corner of the envelope.]

### Notes on News from the Nursing World.

### THE BISHOP OF THETFORD AND NURSES.

The nurses belonging to the Suffolk Nursing Association met last Friday at Wolverston Park, on the invitation of Mrs. Berners, one of the members of the central committee. A short choral service was held in the lovely little church of St. Michael's, in the park. An eloquent address was given by the Bishop of Thetford, who having dwelt on the inscrutable mystery of pain, said, that "nurses and physicians who are brought into daily contact with pain and misery must look upon them as enemies, evil powers to be fought and overcome." He described his hearers as "messengers of God." Nurses could be scientific and skilful to the highest degree, but he asked them above all to see, as they stood by each bedside the image of the Lord Himself in each suffering patient. The nurses were served with lunch and tea in a tent on the grass, and during the afternoon enjoyed a delightful walk on the banks of the river Orwell which surrounds the estate.

### A COMPLIMENT TO FLORENCE NIGHTINGALE.

An instance of the honour in which Miss Nightingale is held by all sorts of people was afforded the other day at the Edgware Police-court. The chairman of the bench had before him the case of Bridget Mackenzie, a woman of seventy years of age, who was charged with sleeping in a field without visible means of subsistence, and also with being drunk. It transpired that she had been before the Court several times for a similar offence, but the Chairman said that, "although the old woman was addicted to drink, the fact that she had been a nurse under Florence Nightingale commanded some sympathy." Showing his sympathy in a practical form, he sentenced the culprit to a month's imprisonment as a second-class misdemeanant. Sad as it is to see a woman with an honourable past in such a position, it is gratifying to find that her association in earlier days with Miss Nightingale was not lost sight of by the magistrate.

### THE NORFOLK DISTRICT AND COTTAGE NURSING FEDERATION.

There is ample scope for the operations of this association, which has been set on foot by the Countess of Leicester and other well-known ladies in the county, though, in referring to the fact, "that many prominent people have come forward in a handsome and generous manner," the *Norfolk Chronicle* says, "the list of subscriptions is remarkable for the number of persons whose names are conspicuous by their absence." The objects of the federation must nevertheless commend themselves to all who are concerned to promote the well-being of a section of the community urgently needing help. It is desired to assist poor districts with grants for the training of nurses or other exceptional needs, to supply temporary nurses in the case of epidemics, to lend expensive apparatus, and generally to organise the work of nurses in Norfolk. We can only conclude that want of knowledge of the aims of the movement is the reason why it has failed at the outset to attract the general support it merits.

### UNIVERSITY COLLEGE HOSPITAL.

It is satisfactory to hear that the three concerts which have been held for the benefit of University College have resulted in a sum of £110, of which about £70 will be handed over to the hospital after payment of expenses. The concerts were an experiment, and have succeeded beyond the expectations of their zealous promoters. The nurses of the hospital shared in the good work by selling programmes. The new hospital is fast advancing towards completion, and the wards of the erected wing are in process of plastering. In August the hospital will be closed for a month to allow time for the change in the nursing staff. The "sisters" who have managed the hospital for so long are leaving, and the sister superior will be replaced on September 1st by a matron. Most of the older nurses will probably leave with the sisters, but those wishing to stay under the new conditions are to submit their names to the matron.

### A STRANGER IN A STRANGE LAND.

An army nurse from America, who has lately returned to Washington from Cuba, insists upon the necessity of a nurse who undertakes work in a foreign country endeavouring to obtain at least a slight knowledge of the language before she starts on her mission. She herself found the ignorance of Spanish most embarrassing, and she says it gave her "the blues" not to be able to make even the children—or the cat—understand her. "One day, just before hostilities had come to a definite end, she was startled by the unexpected visit of her Cuban laundress. The woman was intensely excited. She gesticulated and talked. The nurse did not know a word of what she said, but the pantomime filled her with terror. The Cuban's hands seemed to speak of an attack upon the hospital—of wounded men butchered, and nurses cut to ribbons. The nurse was frantic. She must know the worst. In the hospital was an officer very ill with typhoid fever. She knew he understood Spanish. Only in a matter of life or death would she disturb him, but this was obviously a matter of life or death. She led the Cuban woman to the bedside, and there the story was repeated. The officer listened intently. The nurse held her breath. The Cuban ceased. The sick man turned his head on the pillows. 'She says,' he whispered feebly, 'she says the stripes in your pink shirt have run, and she doesn't know what to do with it.'"

### THE NEW NURSES' HOME AT DUBLIN.

A garden fête took place last Friday and Saturday in the grounds belonging to the Hon. Mrs. Millar, Killarney Woods, Bray, near Dublin, with the object of raising funds to furnish the new nurses' home just attached to Dr. Steevens' Hospital. This venerable institution, which has existed for two centuries, has an organised nursing school, but hitherto the nurses, numbering between 50 and 60, have had to seek accommodation where they could get it. Taking advantage of the Queen's Jubilee, the committee issued an appeal for money to build an adequate home, and up to the present a little more than £1,000 has been received. The cost of the new building is £6,000, so there is still an opportunity for the generous to subscribe. Lady Cadogan and Lord and Lady Powerscourt were the patrons of the fête. Ladies resident in the neighbourhood provided attractive stalls, and several artistes gave their services at the concerts.

### A SENSIBLE DIETARY FOR NURSES.

THE Colchester Guardians have recently passed a dietary for their nursing staff which includes a liberal amount of fresh fruit, vegetables, butter, and eggs. The nurses are also to have fish and fowl or game once a week, and the meat supplied is to be varied. Even pickles and table sauces are not omitted in the list, and each nurse is allowed a pound of jam or marmalade weekly. This compares very favourably with the dietaries of the staffs of many poor law infirmaries.

### SIXTY HOURS A WEEK.

AT a meeting of the Bristol Guardians the other day it was stated that "one of the assistant nurses at the Stapleton Workhouse is on duty sixty hours per week." Dr. Hayman said at the same meeting that he did not believe the Guardians would think one assistant nurse and one probationer were sufficient to look after 130 old people at night. He also mentioned that in the male infirmary, with 120 beds, there were only one assistant and one probationer for the insane at night. Clearly, an augmentation of the nursing staff at Stapleton is urgently required.

### BRADFORD ROYAL INFIRMARY.

THE annual day in the country shared by the nurses of the Bradford Royal Infirmary took place on Tuesday and Friday in last week. The chairman of the House Committee, Mr. W. C. Lupton, invited them to a picnic at Weston Moor, and afterwards to tea at Weeton, near Harrogate. The nurses went by train to Otley, where they were met by charabancs, and driven on to the moor. Cold lunch was partaken of at Vavasour Castle, after which the party had a ten-miles drive through the beautiful Washbourn Valley, and through Farnley Park to Weeton. Here they were met by Mrs. Lupton, the Mayor of Bradford (who is president of the institution), and by members of the board of management and the honorary staff. After tea, boating, bowls, and other games were enjoyed. The nurses, in fact, had a most enjoyable time, and thoroughly appreciated the little variation in the daily routine of their work.

### QUEEN CHARLOTTE'S NURSES.

ON July 17th the Duchess of York opens the new home just built to accommodate the pupil nurses of Queen Charlotte's Hospital. It may be remembered that they have for some time been lodged in three houses in Blandford Square, which were very inconvenient and inadequate. Moreover, they are no longer available, as they are about to be pulled down. The new home is a handsome building of red brick, and is nearly opposite the hospital. It consists of four storeys and a basement, and contains about sixty bedrooms. It is in charge of a home sister (Miss Barry), whose appointment was announced some little time ago. The home is not yet open for inspection, but the old furniture has been refurbished and supplemented, and the result is expected to be pretty and comfortable.

### THE SOUTH-WEST HAM HOSPITAL AND NURSING HOME.

THE inadequacy of this little institution to the needs of the great district surrounding it is pointed out by Dr. Margaret Pearse, the lady medical officer in charge. There are 13 beds in the hospital, and more than 130 are required. Only the worst cases can, therefore, be taken, though, as Dr. Pearse says, this means that lives which might be saved are sacrificed. From 8,000 to 10,000 out-patients receive treatment. Miss Tillyard is the matron, and mention of the fine example set by the poorest people in Canning Town may incite others to do likewise. By their pence subscribed weekly they maintain one of the cots.

### ROBBING NURSES OF THEIR HALF-DAY OFF.

AN audacious attempt was lately made to deprive the nurses employed by the Wandsworth and Clapham District Board of Guardians of their half-day off. It was calmly suggested that during the period of annual leave granted to the nurses the night nursing staff should be robbed of their half-day off. Happily Mrs. Gray came to the rescue of the staff, and, at her instance, the Guardians decided to obtain extra help in the holidays. But it is amazing that the defeated proposal should have been seriously put forward.

### A NEW NURSING HOME AT BARRY.

WITH the rapid growth of Barry the work of the nursing staff has steadily increased, and the new Victoria Jubilee Nurses' Home, which has just been completed, was very badly needed. It is a handsome and commodious structure, and has been erected entirely by means of voluntary subscriptions. Another thousand pounds is, however, required to defray the expense of furnishing, &c., and this debt the promoters hope to wipe off shortly.

### A PUBLIC BENEFACTOR.

MR. A. H. MONCUR, the donor of a sanatorium for the treatment and cure of consumption at Dundee, is a large employer of labour in the town. He was greatly impressed with the fact that one in every ten deaths in Dundee was caused by consumption, and that about half of those who died were mill and factory workers. Mainly in their interest, the ex-Provost came forward with £10,000 to provide them with an institution which is usually only within the reach of the well-to-do. The sanatorium is delightfully situated, and the terms of residence—thanks to Mr. Moncur's munificence—are of the easiest. This is only his latest act of philanthropy, for he was one of the founders of the Sailors' Home, the Night Refuge for the Homeless, and the Sick Poor Nursing Society in Dundee.

### THE NURSES' HOSTEL.

IT is not always an easy matter for a nurse to obtain a night's lodging in London. A nurse often arrives late in the evening, and, after delivering her patient safely at home, does not know where to find her own bed. It was the knowledge of this practical difficulty that led Miss Catherine Wood to found the Nurses' Hostel in Percy Street. Three years ago pressure on the accommodation resulted in the building of the commodious and convenient premises in Francis Street. It was an enterprise that required courage and faith, for the capital was subscribed by nurses, and its success or failure therefore concerned women who could ill afford to sustain pecuniary loss. The balance-sheet for the third year will shortly be issued, and meanwhile the directors are able to announce that the hostel is an assured success, both financially and socially. The dividend will be larger than hitherto, whilst the number of nurses availing themselves of the hospitality of the hostel has considerably increased.

### NO MALE VISITORS ALLOWED.

THE authorities of the Belvidere Hospital are being very freely criticised in Glasgow owing to the posting of a notice that "no male visitors are to be allowed to meet nurses in the grounds." This is the more curious if, as it is stated in one of the Glasgow papers, that the storemaids are permitted a privilege which is denied to the nurses. Of course, it is possible that the regulation, which is new, has been imposed because some nurses have had too many visitors; but, even if this be so, it does not seem fair that all the staff should suffer in consequence. It is true that the nurses go out for two hours in the day, but at a time when their male relatives, or friends, are naturally engaged in their professional or business duties. There must, of course, be restrictions so far as visiting is concerned, whether it be that of brothers, or cousins, or gentlemen who want to be nearer relatives still; but that the Belvidere nurses should only have the chance of seeing their male friends once a month is, on the face of it, rather tyrannical.

### FLAT-FEET.

OBSERVING that skipping, as an exercise to prevent flat-foot, was advocated in "The Mirror," a correspondent asks to add another hint with regard to the cure, or, at any rate, alleviation of this painful and frequent complaint. Our correspondent says: "To those who could not or would not skip, I suggest simply trying to stand on tip-toe whenever possible. Even a few minutes spent in this way every day will do wonders, especially in the early stages of the trouble. A nurse, a friend of mine, saved her feet entirely from becoming flat in quite an unexpected way. After she had been a few months in a hospital she was placed on night duty in a small ward to attend to some simple case. Immediately under the ward was the matron's bedroom, and so afraid was my friend of awakening her that she walked on tip-toe whenever she had to move about. A fortnight of this quite cured her feet, which were threatening to 'give way' before she was put on to night duty. Walking on tip-toe strengthens the weak muscles. For the same reason bicycling is good, and dancing would have a similar effect. Bathing the feet and ankles in warm water for ten minutes every night was also recommended to me by a famous orthopædic surgeon as treatment for 'nurse's feet.'"

### FETE FOR THE BENEFIT OF INCURABLE CHILDREN.

THE sale of work and garden fête at the Hospital and Children's Home, Maida Vale, on Friday, was opened by Lady Grant Duff, who made a gracious little speech. Canon Duckworth welcomed her, and the Chairman (Major Deane) thanked her for coming. In the day room several lady patronesses were introduced by the lady superintendent of the home, Mrs. Bruce. The garden was gay with the stalls, each shaded by a gigantic umbrella, draped in green muslin, and heaped up with pretty things. The ladies serving at the stalls wore badges of different coloured roses, whilst the crippled children had one composed of daisies. Stall I. contained the work in wood-carving, bent ironwork, fretwork, needlework and knitting, of the children, and some of it was quite wonderful. It was all sold, and a fourth of the price of each article was added to the worker's savings in the bank for his or her own use. The flower stall was very beautiful, and Miss Lily Grant Duff was amongst the saleswomen. Several well-known artists contributed to the picture stall, and many charming things in the way of frames, fans, china, and glass adorned the other stalls. It is estimated that about £120 was realised.

### SHORT ITEMS.

THE Duchess of Albany attended the opening of the Shaftesbury Institute last week, and presented to the deaconess a banner as a memorial of the gift of £1,000 by Miss Barclay to provide the new residence.—Lady Hermione Blackwood, daughter of Lord Dufferin, who was nursing for some time at Chelsea Infirmary, has entered the London Hospital as probationer. Lady Dufferin's interest in medical work has doubtless satisfied Lady Hermione of the importance of a technical knowledge of nursing in organising relief for the needs of the sick on a large scale.—At the annual general assembly on Saturday of the members and associates of the Grand Priory of the Order of the Hospital of St. John of Jerusalem in England, an interesting feature was the mention of the enrolment in the Order, at the instance of the India Office, of a number of officers in the Indian Medical Service as honorary associates, and of nurses as honorary serving sisters, in recognition of devoted work during the plague epidemic.—At the Royal National Hospital for Consumption at Ventnor (of which the Queen opens the Battenberg wing during the summer), the patients were agreeably entertained in the grounds last Friday by the Royal Victoria Minstrel Troupe.—Mr. Parker Smith postponed the questions which he was to have asked the Lord Advocate on Monday about the case of Mary Carroll, who died in Duke Street Prison, Glasgow, under peculiar circumstances.—An extremely successful carnival was held in the Clifton Zoological Gardens last week for the benefit of the new nurses' home connected with the Bristol Royal Infirmary, now in course of erection. About 15,000 persons attended, and it is believed that the net profit will approach the handsome sum of £1,000.—Lord James of Hereford will distribute the prizes to the students at Charing Cross Hospital on Wednesday afternoon next, at half-past three.—On Tuesday the annual sale took place at the Royal Hospital for Incurables, West Hill, Putney. The Duchess of Sutherland performed the opening ceremony and then visited all the stalls, making many purchases. The work is chiefly done by the patients themselves, and includes a large quantity of well-made underlinen, aprons, plain and fancy, children's frocks, table centres, tea cloths, woollen shawls, &c., &c. There were many purchasers, and the receipts were highly satisfactory.

### Gynæcological Nursing.

By G. A. HAWKINS-AMBLER, F.R.C.S., Surgeon to the Samaritan Free Hospital for Women; Assistant Surgeon to the Stanley Hospital, Liverpool.

(*Continued from page* 168.)

### ANTISEPTICS.

THE nurse who has received a general training is, of course, familiar with the necessity for the general use of antiseptics in modern surgery, and the various methods of using them. In no branch of surgery are they more important than in the practice of gynæcology, and it will be well in this place to briefly recapitulate several facts connected with this branch of our subject.

It has been shown that the air contains large numbers of living organisms of a low type of life, many of which are harmless, but some varieties extremely dangerous, not only to the success of any operative procedures but even to life itself. These organisms are so minute that they are not merely invisible to the naked eye, but require powerful microscopes to demonstrate their presence. If you can place a trifle of 250 millions of these germs on an ordinary postage stamp, and reflect that they multiply with the most extraordinary rapidity, it will be easily understood that mere cleansing, as practised by the housewife, however scrupulous she may be, is of no avail for the removal or destruction of these creatures. They exist in the air, on the vessels and instruments used, in the skin of the hands of the surgeon and of the nurses, and in that of the patient. If they gain admission into a wound or into a susceptible part of the body in any quantity, it has been shown that not only do they delay the normal healing processes of an injured tissue, but they produce inflammation of a more or less serious character, and sometimes so serious as to destroy life. The fact that these germs may be separated and cultivated in certain materials, and inoculated in the system with the production of very definite results, is sufficient to show that we are not dealing with any imaginary enemy or trifling with any mere scientific fad, but that we are of necessity to take such steps as will prevent the inoculation of our patients with organisms of a very deleterious character. Provision is made in the body for the destruction of these germs. Certain blood cells have been shown to have a very powerful action in destroying them and the products of their life and activity; but you cannot always guarantee that these cells—the police of the body—will exist in a sufficiently healthy condition, or be available in sufficient numbers to cope with large quantities of germs in any particularly active condition. We cannot, then, depend on the tissues of the body for dealing with these injurious organisms, but it must be our care to exclude them altogether or as completely as possible, or at least to ensure that those gaining access to the tissues shall have their activity damaged by the means at our command.

The ideal operation is an aseptic one, that is, an operation performed under conditions which prevent the access to the tissues of noxious germs. The study of their life history has shown us that this can be obtained in various ways. Asepsis prevents the admission of germs. Antisepsis destroys the germs and their poisonous products, and when either of these processes is properly carried out, the risks of any operative procedure and the pain and trouble connected with it are either entirely eliminated or very considerably lessened.

The most efficacious manner of dealing with microscopic organisms is to boil them. We may take it as proved that instruments, for example, boiled for from fifteen to twenty minutes in water containing 5 per cent. of carbonate of soda will not only be free from living germs, but from the spores of these germs, which are sometimes more difficult to destroy than the parent germ. A surgeon has, in these means, a reliable and easy method of securing surgical cleanliness. Instruments so treated are described as being sterilised, that is, they are freed from germs that are living and have the power of multiplying. In the same way we find that boiling water for fifteen or twenty minutes will destroy the germs it contains, in short, sterilise it. But it is not always possible to boil everything that is brought in contact with the patient, and we can only secure asepsis in such cases by a prolonged process of cleansing, and by the use of antiseptics. An antiseptic in common use is carbolic acid in solutions of 1 in 20 to 1 in 40 parts of water. It is, perhaps, the most universally useful antiseptic. Its action is penetrating, it will destroy most germs, and it gains access to them more freely than is the case with other materials. The trouble about carbolic acid is that it is extremely irritating to the hands, and, of course, to the tissues, and, like other antiseptics, being poisonous and irritating, it cannot be used for abdominal operations, at least inside the peritoneum. For a vaginal douche we should use carbolic acid to the strength of 1 in 40 to 1 in 60, that is, an ounce to every two or three pints of water, and for use within the uterus of the strength of an ounce to about two quarts of water. It should be thoroughly mixed and stirred into the water, as it frequently settles to the bottom of the vessel. Many alternative antiseptics are used, of which we might mention two or three. Corrosive sublimate of a strength of 1 in 1,000 to 1 in 3,000 parts of water is a favourite antiseptic, but it is not so penetrating as carbolic acid, and it damages instruments. It is irritating in strong solutions, but it is less harmful to the hands. Many surgeons use preparations like Jeyes' Fluid and Lysol, which are very efficacious, but have the objection that both make so cloudy a mixture with water that it is difficult to find instruments that are covered with them, and Lysol is very soapy and unpleasant to work with.

Tincture of iodine of a strength of one or two teaspoonfuls to a pint is a valuable antiseptic, and chinosol is very powerful, but it is apt to blacken instruments. And so we might run through the multitudes of antiseptics which flood the advertisement sheets of the medical papers, and appeal some to one surgeon some to another. But the surgeon in charge of a case will indicate the particular preparation for which he has a preference and the strengths in which it is to be used.

### Cases of Interest.

### SWALLOWING A CHAIN.

A NURSE mentions the case of a steel chain having been swallowed by a boy aged two and a half years in the county of Oxfordshire. Castor oil was prescribed and given. The child suffered very little pain, and after seven days passed the chain intact, but much discoloured. The dimensions of the chain were as follows: Length, 6 in.; weight, ¼ oz.; number of small links, 21 (each ¼ in. wide); number of oblong links, 2 (each 1 in. long).

### Where to Go.

HIGHBURY ATHENÆUM.—July 8th, 4 to 7 p.m. "Children's Fair in Flowerland," with sale of work, in aid of the London School Nurses' Society, to be followed by demonstration classes in the lower hall. Canonbury, Highbury, or Mildmay stations, North London line; Green Lanes tramcars, and Highbury omnibuses.

### The Ideal Matron: What She Needs to Be.

A CHAT WITH MISS LÜCKES, MATRON OF THE LONDON HOSPITAL.

Just as the position of general is the goal of most young men who obtain a commission in the army, that of matron in one of the great hospitals is the desire of most young women who enter the nursing profession. Moreover, if the latter are inspired by the enthusiasm for humanity which in the case of a nurse is as much needed to ensure conspicuous success as courage and capacity in that of a soldier, they set before themselves a high ideal. How is it possible to achieve such an ideal? This is the question which on the occasion of an interview with Miss Eva Lückes last Friday, I asked the matron of the London Hospital, with her unique experience and knowledge, to answer.

"It is not easy," Miss Lückes said, "to give you a reply. But, of course, it is indispensable that any woman who aspires to be an ideal matron should, to begin with, have the best possible training."

"You take special precautions here to ensure that result?"

"Yes, candidates who wish to become regular probationers have to undergo a preliminary training lasting for a period of six weeks. At the end of the six weeks examinations are held. The main object of this training is to test the character of the applicants. It seems only a fair thing to do. There are 28 pupil-probationers, and they are under the charge of an experienced sister, who gets a very good notion of them while they are at Tredegar House. Dr. Warner sees all our candidates after I have seen them before we apply for references. It would be a waste to give them the training unless there was a reasonable prospect of success."

"In what are they instructed?"

"Chiefly in such household duties as they will have to perform when they are admitted to the wards. They get used to wearing the uniform, observing punctuality at meals, working up to time, making beds, and to everything practical. We teach them also to pad splints, to take temperatures and to chart them, to bandage, and to learn the use of all the little instruments employed in a ward. In fact, whatever will help them to know the right way of doing routine duties for patients—that is the great point in view."

"And the lectures?"

"There are twelve lectures on each subject taught during the six weeks. The pupil-probationers come to the hospital to have lessons in sick-room cookery. After each set of lessons practical and theoretical examinations are held by examiners from the National School of Cookery. We give a little certificate for that if they obtain the full certificate."

"Of course, they are not all found suitable for the profession?"

"Some few are hopeless, and do not come to the hospital at all. Others are doubtful, and I put them for the month's final trial, which they have to undergo here, in the wards where I am sure I can get an opinion about them on which I can rely."

"Does that month count in the training?"

"Yes; the two years date from the time they enter the hospital. You know our standard is that laid down originally by Miss Florence Nightingale. We have three courses of lectures. I take the general details of nursing, the surgeon takes elementary anatomy and surgical nursing, the physician elementary physiology and medical nursing. We have also an outside examiner, appointed by the committee, who sets the questions for the examination, and the papers are forwarded to him. After the third set of lectures we have the final examination, which is not only written but *viva voce* and practical. In other words, the probationers are examined, as far as possible, in technical knowledge; while, in order to make the theoretical training more thorough, everyone going in for the next annual examination has one instruction class and two study classes each week."

"How many probationers attend the instruction classes?"

"Only six. That makes the teaching individual. The class sister corrects the notes on the lectures, and each month a written report of the progress made is given to me. Probationers may come in the middle of a course of lectures, but they must have the full term, and thus it sometimes takes two years to get the one year's theoretical training. But time is only a single element; you cannot make a nurse in a given time. Moreover, more experience can be gained here in a few months than in two or three years in some hospitals. The engagement of each probationer to serve the hospital is for three years, but we deem our nurses qualified to nurse after two years' training because of the opportunities we offer, and the varied experience which we insure to every probationer."

"What are the salaries?"

"For the first year £12, for the second £20, and that for the third depends upon what position they are appointed to fill. The staff nurses begin at £24, and rise to £27 at the rate of £1 a year. Our private nurses receive £30 the first year, £35 the second, £40 the third, and £45 afterwards. The salaries of the sisters range from £30 to £60. All of them have half their annual premium to the National Pension Fund paid so long as they are in the service of the hospital if they join the Fund. The sisters are a very small proportion to the others, because they are in charge of so many beds. We think thirty about the right number, but it is very common for a sister to have fifty-four or fifty-nine. I heartily wish this was not necessary. It is very good training for matrons, but it does not make it easier to manage the work. But we cannot alter the way the hospital is built."

"When a private nurse returns from a case does she have a rest?"

"Yes, for a day or two. Then she takes special cases in the wards, or anything that she is needed for. Private nurses have holidays, which often accumulate, at the rate of a whole day once a fortnight. They are on duty as extras when they have had their holiday."

"As I suppose the most ardent enthusiast would not object to holidays, and would like to be off duty some time, perhaps you would tell me your rules in regard to them?"

"Our chairman, Mr. Sydney Holland, has very strong views on off-duty times. Probationers on day duty get a whole day off duty each fortnight; nurses and probationers on night duty a day and a night; staff nurses on day duty half a day every week, and a Sunday off every four weeks, or if they like to have their half-day on the Saturday before the Sunday they can go away for the night; sisters have half a day every week and every other Sunday, with a night out once a fortnight if they choose; all our nurses get two hours off every day in the daylight, so that they can always go out. No exceptions are made to these hours."

"As to holidays," continued Miss Lückes, "probationers have a week at the end of each six months, and a clear month at the end of the second year, before being appointed on the permanent staff. Staff nurses have three weeks leave annually, and sisters a month. We should like them all to have a month, when we have enough nurses. Everything approaching sick leave is extra and does not count in the holidays."

"Would you like to say anything about the night nurses?"

"Owing to the number of acute cases we have an unusually large proportion on night duty. Our wards are in sets of four, and a night nurse and two probationers are at one end of the division, with about 28 patients. Probationers are put on for three months' night duty, and we consider this very valuable training."

"You have no permanent night nurses?"

"No, it is very many years since we had any. I greatly disapprove of permanent night nursing. It is bad for health, and the nurses get slack in their work. Our staff nurses take three months' night and three months' day work alternately. In this way the patient has the same nurse, and the ward does not suffer by the staff nurses changing for day or night duty because the sister is responsible."

"There is a great deal in what you have said to afford nurses who aspire to be ideal matrons food for reflection, but I should like some further light on the subject."

"You hardly need to be assured that the aspirant should aim at going through the different posts as best she can. But I think that nurses often fail to take enough trouble about the domestic side of the work. I mean the linen-room, the servants, the housekeeping part. A knowledge of these things does not come by instinct, and the management of household matters in an institution is a little different from ordinary home housekeeping. I generally have some holiday sisters, and I give them, if I can, three months as assistant sister in the Nurses' Home to obtain their experience. Perhaps the best experience for the discharge of the duties of matron is three months' business training in my office. I always have one, and often two, holiday sisters, who write letters, deal with forms of application, see visitors, show people over the wards, and answer inquiries. Of course, training in nursing is the first thing necessary for a matron. I have only trained nurses in all the responsible appointments in a hospital—in the office as well as in the supervision of the Nurses' Home."

"Your point, I gather, is that experience should be gained in every department?"

"Precisely—in every department that a matron has to take charge of. Here there are two matron's assistants. One looks after the office, the other after the household, and both share the supervision of the wards. In an ordinary sized hospital one assistant matron helps the matron in everything. A nurse should, if possible, be an assistant matron before she becomes a matron. All find that a little practical training is an advantage. Moreover, you cannot train matrons wholesale."

"That is, there are women who are naturally disqualified to be matrons?"

"Undoubtedly. For instance, it is not the *first* qualification for a matron that a woman should 'know how to put her foot down,' though that is useful to a certain extent. It is of vital importance that she should be a judge of character. She should not be a martinet, but an unselfish, tactful woman; firm, yet gentle; careful about the observance of rules, but while conversant with all details, able to avoid troubling herself over-much about them; in fact, she should be like the mother of a household, adapting herself to the peculiar circumstances of the institution over which she presides, and the class of workers whom she controls."

Then, having taken up more of Miss Lückes' time than I anticipated, Sister Sarjeant was good enough to show me over some of the wards and the Nurses' Home, with its daintily furnished sitting-room, its useful sick-room, and its admirable appointments throughout. The London Hospital is like a small city, and its staff amounts in number to a small army. But a passing glance suffices to confirm the opinion that inside its walls there are all the opportunities for graduating in the studies, for acquiring the experience, and for building up the character, which the nurse who hopes to become a matron—even an ideal matron—can desire to enjoy.

### International Congress of Women.

THE proceedings of the International Congress of Women began with the welcome of the delegates on Monday afternoon, a most interesting meeting, at which some 28 nationalities were represented by their most prominent women, the chief feature being Lady Aberdeen's presidential address. Business began on Tuesday morning, and meeting follows meeting throughout this week at Westminster Town Hall, the Church House, and St. Martin's Town Hall. To summarise the discussions chiefly interesting to our readers which have already taken place: "The Life and Training of the Child," on Tuesday, brought together large audiences, and many good papers and speeches; "The Teaching of Defective Children" being treated by Dr. Francis Warner and Mrs. Burgwin. Mrs. Ellen C. Johnson and Madame Bogelot read papers on "The Treatment of Women in Prisons" in the Social Section; and "Medicine" was the subject of the afternoon debate in the professional section, Mrs. Garrett Anderson presiding. Mrs. Scharlieb gave an admirable account of the present position of medical women in Great Britain. Mr. Wallace Bruce, chairman of committee of the Association for Promoting the Compulsory Registration of Midwives, said a few timely words in reply to some remarks from Dr. Sarah H. Stevenson, U.S.A., which showed that the midwife question as it exists in England to-day is little understood by foreigners.

On Wednesday morning in the professional section the work of women inspectors was discussed, and the accounts of the progress made in their own countries in this connection given by ladies from Canada, the United States, Holland, &c., proved exceptionally interesting.

The foreign medical delegates were entertained at the new Hospital for Women after the meeting on Tuesday, and Mrs. Heywood Johnstone had a pleasant gathering of medical and nursing members of Congress at her house in Draycott Place. Miss Norrie, from Denmark, and Fröken Gertrud Adelbourg, from Sweden, spoke, and a nurse from Kimberley gave some striking details concerning nursing in South Africa.

### The Nurses' Quarters at the Royal London Ophthalmic Hospital.

No pains have been spared to provide for the comfort and welfare of the nursing staff at the splendid new Royal London Ophthalmic Hospital opened on Tuesday by the Duke and Duchess of York. The nurses' quarters are on the third floor of the building. Separate bedrooms, of a good size, are provided for each nurse and probationer, and their arrangement as to furniture is really ideal and worthy of all praise. The fittings are of polished pine, specially designed as fixtures, affording a maximum of accommodation and occupying a minimum of space. Hanging cupboard with long glass, a generous supply of shelves and drawers, special cupboards for hats, boots, and bottles, bookcase and washstand, surround the fortunate nurses who will inhabit these pleasant rooms with all a woman can want for her toilet comfort. Miss Robinson, in thinking of these material goods, has not forgotten to please the eye, and the dainty bed-spreads in two shades of green and the specially-made strip of green carpet are charming. There is a cosy sitting-room with settee seats and inviting easy chairs, a good-sized dining-room, and sick quarters on a top floor, entirely isolated and self-contained, provided with every requisite for illness. The nurses have an ample supply of bath-rooms, and another of Miss Robinson's special devices is a box-room most conveniently divided into numbered compartments to correspond with the rooms. The matron, architects, and those who have so well carried out their ideas and plans are to be congratulated upon the result.

### A Year's Plague Nursing in India.

By a Sister.

### FIRST IMPRESSIONS.

It was merely by chance that I took up a copy of The Hospital when I was on a visit in Devonshire, and in its columns I saw an article in which nurses were asked to volunteer for plague work. Rules and conditions were given and details regarding salary. This was just the opportunity I wanted, as I had been reading with the deepest interest of the terrible time of plague in India, and had heard that some nurses had already been sent out. I accordingly applied at once to the Secretary of State at the India Office, enclosing my certificates, testimonials, &c., which were satisfactory, and was informed that in the event of passing a medical examination I should be accepted, and must in that case be prepared to sail for India at a week's notice. About a month later I received a telegram asking me if I were willing and ready to start by the end of the week. As far as getting my few unsuitable goods and chattels together I thought I was prepared, and it seemed an easy matter to get some ready-made clothes, but alas! for trying to buy light summer things in the month of November. I flew from shop to shop during the few days I had to prepare, and was met with a blank look of astonishment wherever I asked for white muslin blouses and white skirts! Of course I had no time to get them made, so after unearthing all my summer clothing I selected the lightest and coolest, and packed them up with the consolation that others could be sent to me subsequently. It was, however, with a very natural feeling of sadness at the thought that perhaps I was never to return, that I started off on November 25th, 1897, to begin my work of plague nursing in India. At Victoria Station I was glad to meet ten other nurses, fellow-companions, bound on the same mission as myself, and we soon became friendly. The journey to Brindisi was interesting and amusing. It was very cold, but there was no reason to complain of the lack of foot-warmers, as they were renewed far more frequently than we needed them. We were allowed by the conditions of our agreement to have six hundredweight of luggage, but of course the railway people did not permit us this, and we were rather foolishly and alarmingly surprised when we had to pay for it ourselves, especially when we arrived at Modana—where the Italian officials informed us, with many gesticulations, that no luggage whatever was passed free. However, we had all this afterwards refunded to us by Government. We were very glad to have a good night's rest at Brindisi and a refreshing bath after our long journey. The next morning we sailed in the P. and O. ss. "Himalaya" for Bombay. We were provided with second saloon tickets. The boat was crowded, but we found things fairly comfortable. Why are nurses such bad sailors? I am afraid very few of us escaped seasickness, although we were supposed to have had a calm voyage. The heat in the Red Sea was intense, and I vainly longed for those cool muslin blouses I could not get. At Aden we had to leave the "Himalaya," which was bound for Australia, and all passengers for India were transhipped to the ss. "Shannon." Another week of sea, and then, on the morning of December 12th, we found ourselves steaming into Bombay harbour—truly a beautiful sight. We were rather disappointed that, owing to some mistake, there was no one to meet us, and we were somewhat exercised in mind as to what we should do. Fortunately, among the people who were waiting the arrival of the passenger boat were some old friends from our own hospitals also on plague

Plague Camp at Poona.

service, whom we never expected to see. It being Saturday, all the offices were closed at two o'clock, so there was nothing else to do but go to the hotel and wait patiently until Monday before presenting ourselves to the Surgeon-General, who would give us our orders. We did not waste much time, but went about seeing all the charming sights, which we thoroughly enjoyed. On Monday we had orders to proceed to the Surgeon-General's office. We were very kindly received, and, to our gratification, were informed that we might spend a few days in Bombay to do any necessary shopping and to provide ourselves with uniform. All the English plague nurses wore Indian nursing service uniform, which is white, with scarlet collar, cuffs, and waistband, and Sister Dora caps, very pretty and cool. The subject of "inoculation" was broached to us, but we did not avail ourselves of the opportunity then, as we had heard some very foolish and exaggerated accounts of its after effects. Of course, this was a mistake, as I certainly think it would have been wiser had all the nurses been inoculated before entering on their plague duties instead of afterwards. Besides being considered a safe thing to do, it is an excellent example to the natives, who had a terrible dread of it at first, and later on, as we gained further experience, we found that if persons did get plague after inoculation it was usually of a mild type. I will describe my own experiences of being inoculated in a future article. Towards the end of the week four of the nurses received orders to remain in Bombay, two to proceed to Satara, and five to Poona. I was among those sent to Poona, and was very eager to begin my work and to actually see a plague camp. One could hardly realise in merely visiting Bombay that it was a plague-stricken place. Things seemed very prosperous and flourishing all around, and as the English and native community live so entirely apart they are brought very little in contact with each other. The climate of Poona in the winter is really lovely. The mornings are quite cold and fresh, and it is pleasant out of doors all day, in striking contrast to the nasty, hot, sticky climate of Bombay, which, even in December and January, is not pleasant. At this time Poona had been suffering cruelly from the ravages of plague, which showed no signs of abating, and we had the largest plague camp in India, the patients numbering about 500. The illustration represents a view of the camp.

The wards or sheds were built simply of matting held together with bamboo. The ceilings projected beyond and were not attached to the side walls, admitting free ventilation. The same space was allowed between the walls and the floor, the latter being made of beaten-down earth and whitewashed. The beds were of wood with rope laced across They are not sound or comfortable, and are decidedly conducive to bedsores; but most native beds are made in this crude fashion. Our patients had a pillow, sheet, and two or three blankets, and were provided with bed jackets. The rest of the furniture consisted of a table for medicine bottles, another for the dressing-box, lotions, &c., and a wooden box for the ward linen. Earthenware jars containing water were placed round the sheds. These were to be used in case of fire. I do not remember the number of sheds, but they extended over a vast field, and the row of tiny white tents beyond were reserved for cases complicated with erysipelas, and also for people suffering from mania or noisy delirium. The tents were laced down the centre, and I must say it was rather gruesome at night to visit them and to have to unlace them and creep inside. Of course, native assistants were in charge of them. Our medical staff consisted of the superintending officer, one lady doctor, two doctors—one resident—nine qualified hospital assistants, and five dispensers, also resident. The nursing staff comprised 15 English nurses, and about 60 ayahs and 60 ward boys, besides sweepers, &c.

I must confess that my first impression of a plague camp was of a somewhat startling nature, and I wondered how it was possible to do ever so little for such a vast number of people. However, we had to grapple with the situation and to do all we could, and by degrees things began to shape themselves in order and to become easier. We were called at six a.m., and had coffee, buttered toast, and eggs brought to us. We had to be in the camp at seven a.m., at which time the night nurses went off duty. The mornings were very busy. We began by seeing to the general tidiness of the wards, taking of temperatures, washings, and bed-making, which, by-the-bye, was no easy task. I do not know how the natives perform their ablutions in their own houses, but their idea of washing a patient in bed was to sprinkle a few drops of water on their face and also on their bedclothes, and to leave them to dry themselves. They had no idea whatever of the use of a towel. Our willing but ignorant fellow-helpers were also a little uncertain as to the way of making the bed of a helpless patient. They are accustomed to rolling themselves tightly, head and all, in a dirty blanket and lying so all night; but this was not at all suitable for our poor patients, and it was only after many entreaties and scoldings that we persuaded our ward attendants that it was not necessary to drag a patient with a failing pulse and a temperature of 105 deg. off the bed and try to make him sit upright on the floor while they leisurely made his bed. I say "leisurely" because "hurry" is an unknown thing in India, and it was very amusing, but rather aggravating, to see an ayah walking in a slow and dignified way across the ward to fetch something for which you were in a desperate hurry and could have brought yourself exactly twice as quickly. It was some little time before we could rely on our assistants doing as they were told, and it was only with the strictest supervision that we made them at last understand how absolutely necessary it was to be most careful. I have personally seen patients die from sheer neglect in this respect. By nine o'clock the wards were straight. We then returned for a few minutes to our own particular shed to partake of sandwiches and coffee, and soon after the doctors arrived to make their respective rounds. All the sheds were next visited, patients examined, further orders given, and we had not a minute to spare until twelve o'clock, when all the nurses left the camp and the native assistants were solely in charge. We breakfasted at half-past twelve, or rather we had a substantial lunch, and at half-past one six of the nurses returned to duty until four o'clock. Then not only had we our own wards to look after but also those of the six other nurses who were "off" duty. The task was not easy and not very satisfactory, but some nurses wisely had slates hanging up in their wards stating the number and treatment of any special cases. This was a great help, and we were able to do all we could for them, as it was quite impossible to give each case individual attention. By four o'clock we had taken the afternoon temperature in our own wards, given all medicines up to that time, and were relieved by the other six nurses who came on duty until seven o'clock. They continued the treatment and, in fact, did identically the same work we had previously been doing, devoting most of their attention to their own particular wards.

(*To be continued.*)

### To Nurses.

In order to increase and vary the interest in the *Mirror*, we invite contributions from any of our readers in the form of either an article, a paragraph, or information, and will pay a minimum of 5s. for each contribution. All rejected manuscripts are returned, and all payments are made at the beginning of each quarter, *i.e.*, January 1st, April 1st, July 1st, and October 1st.

### Dispensing Up to Date.

By a Nurse.

When I left my hospital a few months ago I made up my mind I would learn dispensing thoroughly, for nowadays the more strings one has to one's bow the better, and I know this is one which stands a nurse in good stead.

I had not the least idea how to set about it, so perhaps my experiences may be of use to others placed as I was then. After spending some little time in searching for a good teacher and place of instruction I decided upon a laboratory in the West-end of London. I found there were several places to which I could go and had no little difficulty in making a choice.

One can go to a qualified pharmaceutical chemist (there are a few lady chemists who take odd pupils, but most of them prefer the regular three years' apprentice, who is destined for the Pharmaceutical Society's examinations), or there are one or two coaches who have qualified teachers and good laboratories, in whose hands one can safely place oneself to gain a thorough knowledge of dispensary work, and—also most necessary—materia medica, chemistry, and pharmacy.

It was to one of the latter I went. On arriving I was ushered into a large laboratory, where about twelve or fourteen ladies were busy at work. A portion of a bench was allotted to each; all round the room were shelves containing multitudes of interesting—and uninteresting—looking bottles. Bottles of every shape, size, and description. I was told off to a place, and then the principal came up to me, dictated a prescription, and showed me how to proceed to dispense it. The first thing I made was some powders, which were nice to do, and very straightforward, requiring only great care and delicate manipulation. I found the apothecaries' scales very difficult to manage at first, and felt clumsy with them through several lessons.

Of course, as in everything else, practice makes perfect, and to become a neat and accurate dispenser you require a great deal of practical work. I felt terribly slow in the beginning, and as accuracy is the chief thing to be aimed at, my teacher did not "hurry me up" at all in the early lessons. Afterwards, it is of exceeding importance to be quick.

Most of my colleagues in the laboratory were working for the Apothecaries' Hall assistant's examination. For this, in addition to unlimited practice in dispensing, they had lectures in materia medica, and pharmacy, and also demonstrations in chemistry. It takes from four to six months to prepare for this examination, but personally I do not think anyone should undertake a post as dispenser, except under another, for a much longer time than that. One should try after having passed the examination to secure several months' practice under strict supervision before taking upon one's-self such grave duties, for life and death are in the hands of the dispenser.

I enjoyed my lessons and the lectures exceedingly, and found the work most interesting, though the amount of dry facts to be committed to memory is rather a tax. Some knowledge of Latin is, of course, necessary, though prescriptions are generally written in "dog" Latin, and to have studied botany would have been a great help to me. I found the materia medica far less difficult to grasp than I had imagined. It is necessary to read up a good deal of chemistry, and the tests must be known. The only really successful way of mastering the latter is by practical work.

Dispensing is far more difficult and serious work than many imagine. True, the compounding of a simple mixture, or the making of an inert ointment requires no very great skill or brain power, but these are examples of some of the very easiest preparations that come in one's way, and the difficult ones are almost unlimited. Those who intend to make dispensing their life-work should, if possible, go in for the Pharmaceutical Society's examinations, and at any rate they should try for the "minor," which requires that candidates shall have dispensed for three years before presenting themselves for that examination. The Apothecaries' Hall assistant's diploma is very valuable to a nurse who aims at cottage hospital work, or to a nurse-missionary going abroad. Here, again, I must repeat how necessary it is to have a great deal of practice, under supervision, before undertaking the sole management of a dispensary, however small.

A good knowledge of chemistry is most important for the aspiring dispenser, or the disasters in dealing with "incompatibles" may be terrible. For instance, whatever is incompatible with opium or arsenic, if not treated carefully, will throw down a precipitate of either of those poisonous drugs, and therefore the last dose in a bottle of medicine, not properly dispensed, might very easily cause a death. I have a vivid recollection of trying to mix a preparation of iron with mucilage of gum acacia, which resulted in the production of a horrible and utterly unmanageable mass, not unlike a sponge-cake!

Almost everything in a dispensary is made with a pestle and mortar, but the ways of manipulating the pestle are various. Pills require a good deal of care, especially with regard to the movement of the pestle. One soon becomes skilful in using the pill machine. Suppositories need the greatest attention, or they are fearful failures. I thought blisters most fascinating to make, especially those I had to draw first; as, for instance, a blister for the back of the ear. Emulsions are not easy to mix, and it is most heartbreaking after making what one fondly imagines is a perfect emulsion, and letting it stand an hour or so, to find little globules of oil floating on the top of the mixture in an utterly defiant manner! Phosphorus is one of the most tiresome things to deal with, and if kept out of water for many seconds it ignites.

It is necessary to aim at extreme neatness and order in dispensing. Labelling, too, requires great care and nicety. Every label should have its edges carefully trimmed with a pair of scissors, and the directions written very clearly. A dispenser should write a very legible hand.

I think most nurses, if they can possibly do so, should try to obtain a good knowledge of dispensing. Even if they never have to dispense, the experience gained in a laboratory will be simply invaluable, and the importance of knowing, accurately, the doses of all poisonous drugs, is obvious. None of us has forgotten the sad death of a patient in a cottage hospital not many months ago through an overdose of opium administered by a nurse; while those who have had much experience in dispensing can well imagine the state of mind of the man, in the immortal Bardell v. Pickwick case, who would not be sworn to serve on the jury because he was a chemist, and had left an unqualified assistant in charge of his shop!

### A Private Home for Ladies of an Advanced Age.

A private home recently opened at Gothic House, Hendon, seems likely to meet a want. It is for ladies of advanced age or for chronic invalids requiring more care than they can receive in their own homes. The house is well suited to the purpose, with pleasant surroundings. The principal is assisted in the care of the patients by a trained nurse, and an agreement has been made with a neighbouring medical man to visit them as often as they wish. The terms are very moderate.

### Echoes from the Outside World.

AN OPEN LETTER TO A HOSPITAL NURSE.

THE necessity for the erection of a Savings Bank on such a gigantic scale as the building of which the Prince of Wales laid the foundation-stone at West Kensington last week makes one doubtful whether, as a nation, we are as devoid of thrift as we are generally supposed to be. There will be house room for 4,000 clerks to begin with, and later on, when all is completed, for 7,000, a great number of whom will be women. They are all to dine on the premises, the whole of the fourth floor being taken up with the commissariat department. The men and women will dine separately, and each will have their separate cooking arrangements. It is strange to associate the place where we used to repair to see Barnum's show with such a sober, earnest side of life as "saving up the pence against a rainy day," but, as a matter of fact, the new Savings Bank (which has become imperative because of the pressure on Queen Victoria Street) is to stand on what was once the winter garden of Olympia, known as "The Annexe." In the speeches made on the occasion I was much struck with the Duke of Norfolk's statement that one out of every five persons in the United Kingdom was a depositor, a very large percentage belonging to the working classes. Like the little child who spoke too soon on Saturday, whilst all, in expectant silence, were awaiting the arrival of the Prince of Wales, and whose squeaky small voice was heard distinctly by the Royal party, I too add, "Hooray!"

IT is far beyond most of us to understand all the "ins and outs" of the Dreyfus case, but we cannot help feeling interested in the return of the exile, and the prospect of reunion between him and his devoted, hard-working wife. Had she been content, as some women might have been, to sit still and bemoan her fate, the probability is that Captain Dreyfus would still have been enduring solitary confinement in the awful climate of the Devil's Island. Where he is now is one of the puzzles of the hour, but it is believed that he is on the "Sfax," and the "Sfax" is *somewhere*, though the object of the authorities is to conceal the whereabouts, so as to land him quietly and avoid unseemly demonstrations. Madame Dreyfus is to be allowed to visit her husband twice daily in his cell, but so firmly has she faith in his ultimate liberation that she has taken a villa at Hulgate, it is said, in which to reside with the Captain after his release.

I AM rejoiced to hear that "King John" will be produced at Her Majesty's Theatre in the early autumn. It is years since it has been produced on the London stage, and to see it staged as Mr. Tree is likely to stage it will be a real treat to all lovers of Shakespeare. I am warning you in plenty of time, so that you may not be too extravagant in "outings" during the next few months, knowing that you have something delightful to look forward to. Everything is getting into trim, scenes are being painted, dresses are being made, armour is being fashioned, and parts are being assigned. Mr. Tree is anxious, if possible, I am told, to secure a real boy to represent Prince Arthur, so that all shall be as ideal as possible.

THE great reduction in omnibus fares in many parts of London will be much appreciated by all of us—as long as it does not "sweat" the drivers and conductors—and not least by nurses, whose recreation frequently consists in a ride on the top of a "'bus," when they feel they *must* have a breath of fresh air and yet their legs refuse to carry them far enough to get it. The Atlas and Waterloo omnibuses have quite a long list of distances which can be compassed for a halfpenny. From St. Thomas's Hospital that modest little coin will take a traveller to Charing Cross, to the Elephant and Castle, or to Waterloo Station, and the penny rides are tremendous, one of the most surprising being from Charing Cross to the Elephant and Castle, or from Baker Street Station to Piccadilly Circus.

THE question of cheap fares on omnibuses makes one think of the poor horses, who really suffer to a distressing amount in the hot weather, working all through the heat of the day. One day lately a fine young horse, I am told, fell dead in the City from the effect of the sun, and it seems sad that, owing to a stupid insular prejudice, little or nothing is done to help them. You must all be familiar with the kind of "horse's hat" which is worn in the south of France, because a picture of it appeared in "Travel Notes" quite a short time ago. The price is only about 1s. each. The hats could all be made by the blind, which would be a means of helping a most deserving class; and the horses, after a temporary resistance at the strangeness of a headgear to which they are unaccustomed, would soon learn to be grateful for the welcome shade. Then what stands in the way of the universal adoption of hats for horses? Nothing, I believe, but the fear of the jeers of the multitude. The first time a lady was seen astride a bicycle she was hooted at all down the street. Had she given up in consequence, perhaps thousands of women would have been deprived of a most healthful form of amusement. In the same manner, if a little more pluck were shown by the lovers of animals, the few—the *very* few—hats which were observed on Monday would soon be followed by the appearance of thousands in the thoroughfares. If the Princess of Wales would only drive down Piccadilly on a hot day with two bonneted steeds it is quite safe to assert that the blind workers would be unable at first to meet the enormous demand for horses' hats which would be at once created.

Do you see that a New York magistrate has been getting himself into dreadful hot water because he has been remarking that nine-tenths of the women complainants who come to his court are liars, and incapable of telling the truth if they tried? I do not wonder that a good many women took up the cudgels and wrote to defend their sex, but they may be quite sure that their protestations were all wasted. A man who is convinced that women cannot be veracious under any circumstances would not suddenly become converted to a contrary opinion because he received a sheaf of letters, terming him, directly or indirectly, a "brute." Now don't vote me almost as bad as the New York magistrate if I say that I am afraid there is a certain amount of truth in the statement. Feminine witnesses are not half so dependable as men because they get so much more easily excited. The strain of giving evidence is great, consequently they either become so frightened that they do not know what they are saying, or else so elated that they feel they must say something which will cause a sensation. The result is that they weave a web of their own instead of repeating just what they saw and heard, but not with any intent to deceive. It is so simple to paint up a story and so delightful to feel that it really sounds remarkably well. But if women once realised how easy it is after the "painting up" process to drift unwittingly down the slippery path of real black lies I am sure they would pull themselves up short once or twice in the middle of a telling anecdote, and so break through the habit. Nurses, I fancy, are, as a class, more inclined to be quietly truthful than most women, because they have so often to answer "yes" or "no" to a question upon which hangs the issue of life or death, and to describe symptoms in cases when a little exaggeration might result in fatal treatment. So they have, or ought to have, an extra incentive to cultivate the flower of truth. What do you think?

### Everybody's Opinion.

[Correspondence on all subjects is invited, but we cannot in any way be responsible for the opinions expressed by our correspondents. No communication can be entertained if the name and address of the correspondent is not given, as a guarantee of good faith but not necessarily for publication, or unless one side of the paper only is written on.]

### GATHERING UP THE CRUMBS.

"A Looker-on" writes: A curious incident occurred in a Poor Law infirmary a short time ago. The matron, walking through the wards (male) of the infirmary with several members of the Board, saw a small pile of dust and crumbs which one of the men had swept behind the day-room door, intending to gather it up when he found a shovel. But the matron was a little too quick for him; she picked it up herself and put it in an envelope to present to the Guardians at the next Board meeting, in order to prove that the wards are not kept clean.

### READING SOCIETY FOR NURSES.

"L. G. M." writes: I have received several answers from nurses who would like to join a Reading Society. But one of these points out that it would be expensive to buy a number of new books, and suggests an augmented subscription, with the idea of getting the books lent. I have been to see one of the leading medical booksellers, and he thinks it might be possible to arrange a lending library for nurses if a sufficient number of nurses joined to make it worth while. Another nurse proposes that one new book should be suggested for reading every quarter only, and questions set on different parts of it every month. Will those nurses who are interested in the scheme communicate their view to me? Then, should the lending library scheme meet with general approval, I would make further enquiries as to charge for subscription, &c. Also may I point out that all communications should be addressed to Miss Moberly, 24 (not 14, as previously stated), Portland Place, S.W.

### THE OPEN-AIR SYSTEM FOR THE NURSING OF PHTHISIS.

"A Constant Reader," writing from the county of Durham, says: I should like to tell you how much I enjoyed reading the four articles on "The Open-Air System for the Nursing of Phthisis," and would be glad to express my gratitude to the writer of them, if I may, through your columns. It is a subject I am greatly interested in, and one of moment to the public in general. I think, if it could be more generally instilled into people how much infection is spread abroad through the terrible habit so many men have of spitting anywhere and everywhere, we should have fewer cases of phthisis particularly in crowded cities. Nurses (especially private and district) should be able to do a good deal in teaching all they come in contact with the necessity of cleanliness on that point. May I add that I look forward to The Hospital each week? It is both interesting and instructive, and keeps one quite *au fait* with the news of the nursing world.

### THE RECOGNITION OF TRAINING.

"M. T. M." writes: The query put under the heading "Recognition of Training" in a previous issue of the "Nursing Mirror" prompts me to write on a matter which, to my mind, calls loudly for redress. A probationer enters hospital for a three years' course of training, very often paying a fee also. She naturally expects to be instructed in the nursing of all diseases taken into the institution during her term there. It only too often happens, however, that she finds herself disappointed. You reply, "It is only reasonable and right that a nurse should be fully trained," but that there is no means of compelling authorities to consider the nurse's training and not their own convenience. That this is so is no less than a huge injustice. One probationer is kept fixed in a certain ward because it suits her superiors. Therefore, she has no chance of nursing any but one form of disease. Another is stuck three out of every four months on night duty, and so sees little of the daily treatment of patients—rarely hears a clinical lecture—and becomes finally so doubtful of her own capacities that when called upon to assist at a serious operation or attend the surgeon in the ward, she feels painfully awkward and inefficient. For my part I only wish that some power would step in to save the nurse from such unjust treatment, and her authorities from the gross dishonesty of launching upon the public a woman whom their certificate alone proclaims to be a "trained nurse."

### "NURSES AND THE OUTSIDE WORLD."

"A Constant Reader" writes: May I offer you my hearty congratulations on the new feature you have recently added to your already useful and interesting paper. I refer to "Echoes from the Outside World," which will, I am sure, be much appreciated by very many busy nurses and probationers who have not the time to read the daily papers or to keep up with the doings of the "world outside" their hospital world. How much I should have enjoyed it in my probationer days! Coming from an intellectual circle, where discussions on the politics of the day formed a part of our daily life, I keenly felt the want of time to read of passing events. This led me, I remember, into disgrace. Anxious to learn how a certain "Bill" was finding favour at St. Stephen's I hastened through "evening work," thus gaining a few spare minutes before "sister" and nurse returned from supper. My fellow "pros." being still occupied in the ward, and lights "turned down," I ventured to borrow a newspaper from a kindly patient, and knelt before the fire to get a gleam of light by which to peruse the coveted sheet. Suddenly an ominous silence made me lift my head, and there, in the doorway, stood sister, looking "terrible things!" My heart sank into my boots, and I felt I had somehow committed an awful crime. After that I got a good-hearted sister to send me an epitome of news at the end of her weekly letter from home. This was in the early "eighties," and before The Hospital came to give us help and amusement. I welcomed it heartily when it first came, and, as a constant reader, now watch its progress with much interest.

### PROVINCIAL HOSPITAL, PORT ELIZABETH.

"An English-Trained Nurse on the Provincial Staff" writes, from Port Elizabeth: I am very much amused at the account of our hospital. In the first place, the nurses are not allowed to run off to the ships every time they come into port. They, of course, go to see their own friends, as any other lady would. We have a staff of twenty-three female nurses, one matron, one night superintendent, four head nurses, five staff, twelve probationers of various grades, two male nurses for the stricture ward, two male wardsmen for the large male European ward, and, of course, the usual porters, &c. There are two resident doctors and a resident dispenser, who live in the cottage supposed to have been turned into a maternity ward. Far from our nurses objecting to the natives, they prefer that ward. For one reason the natives are more easily managed, and for another there is so much surgical work. It would not be possible to keep any ward surgically clean with coloured help. Evidently your contributor has never managed Kaffir servants or she would know how impossible it is to obtain anything like thorough work out of them. Nor does anyone in authority dream of returning to the old state of ward-boy nursing—we might as well have the old Sairy Gamp and Betsy Prigg back again, on the plea that a lady cannot empty a bed-pan. The chronic ward is now under a female nurse, very much to its benefit in every way. The patients (poor old men whose working days are over) are happier and the ward infinitely cleaner and brighter in every way than under the sway of the old wardsman. All the heavy cleaning is done by Kaffir ward-maids, but there is, of course, much that a nurse must do. Our head nurses are all certificated and thoroughly-trained gentlewomen; also several of the staff-nurses are the equals of London trained nurses, and better suited to this country. The daughters of our best Colonial families now take up nursing, and thetone of our large hospitals is quite up-to-date, though the discipline is not so rigid as in England.

### The Royal Maternity Charity.

INSTITUTED in 1759 for delivering poor married women at their homes, the Royal Maternity Charity has verified the declaration made on one memorable occasion by the Duke of Argyll (the president) that "All London is the Charity's hospital, and every street a ward." The festival dinner in connection with the charity took place last week at the Albion Tavern, Mr. C. BARHAM, C.C., presiding in the unavoidable absence through illness of Dr. Fancourt Barnes (the physician-in-chief). The Chairman, in proposing "Prosperity to the Royal Maternity Charity," pointed out that the charity was probably the oldest, as it was undoubtedly the largest, institution of its kind in this country. From its earliest infancy the charity had received the warm support of members of the Royal Family. The object of the charity was to assist the poorest married women throughout the whole area of the metropolis. Though the charity could not absolutely perform that herculean task, it went a long way to accomplishing it, for on an average no fewer than 4,000 poor women were delivered annually. Altogether, as many as 520,000 had been assisted—enough to fairly populate many a large town. The mortality among the patients was remarkably low, and during the last 10 years, though 40,000 women had been delivered through the medium of the charity, there had been only 55 deaths. An interesting ceremony was the reception of the four principal midwives, who presented a contribution of £7 5s. 6d., being the result of a collection among the 40 midwives who constitute the nursing staff of the charity. The Chairman, in proposing "The Health of the Secretary," testified to Mr. Long's devoted services to the society.

### Appointments.

GRAVESEND HOSPITAL.—On June 22nd Miss Marian Measures was appointed Matron. She was trained at Guy's Hospital (1886-1889), and has since been ward sister at the Kent and Canterbury Hospital (1890-1891); night sister, and afterwards ward day sister, at the Manchester Children's Hospital, Pendlebury (1891-1896); and subsequently assistant matron and home sister of the Seamen's Hospital, Greenwich.

### Minor Appointments.

THE SANATORIUM, DALTON, HUDDERSFIELD.—On June 20th Miss M. Emerton and Miss Isabel Kemley were appointed Sisters. Miss Emerton was trained at the Leicester Hospital, and her appointments have included charge nurse one year at the Western Hospital, Fulham; three years at Hampstead; and nurse in charge of the Throat Hospital, Brighton. Miss Kemley was trained at the Salop Infirmary, and was afterwards sister at the Sanatorium, St. Helens.

EXETER WORKHOUSE.—On June 20th Miss Helen G. Spalding was appointed Superintendent Nurse of the hospital and lying-in wards. She was trained at the Manchester Workhouse Infirmary, Crumpsall, Manchester, and has been nurse at the Heckingham Workhouse of the Loddon and Clavering Workhouse.

GORLESTON COTTAGE HOSPITAL.—Miss Carson was appointed Matron on June 16th. No particulars of training in previous appointments have been sent with the announcement.

WOODFORD JUBILEE HOSPITAL.—On June 22nd Miss L. Stone was appointed Matron. She was trained in the East London Hospital, and has been seven years matron at Brentford Cottage Hospital.

BEDFORD UNION WORKHOUSE.—On June 3rd Miss M. C. Bishop was appointed Superintendent Nurse. She was trained at St. George's Hospital, and has been charge nurse at the South-Eastern Hospital.

### For Reading to the Sick.

"HELPFULNESS."

If there is power in me to help,
It goeth forth beyond the present will,
Clothing itself in very common deeds
Of any humble day's necessity. —*MacDonald.*

Freely we serve
Because we freely love. —*Milton.*

She doeth little kindnesses
Which most leave undone, or despise;
For naught that sets one heart at ease,
And giveth happiness or peace,
Is low esteemed in her eyes.

She hath no scorn of common things,
And though she seem of other birth,
Round us her heart entwines and clings,
And patiently she folds her wings
To tread the humble paths of Earth. —*Lowell.*

By the light of Christian love
'Tis blind idolatry no more,
But a sweet help and pattern of true love.—*Keble.*

One in each interest, hope and fear,
Whatever chance betide;
One in affection's bond—though two
To comfort, strengthen, guide.

When every little fault is seen,
And every fleeting mood,
And all the nobler impulses
Are shared and understood. —*Lecky.*

He sees the gleams
Of better thoughts across the murkiest gloom,
The need of good amid the howling wastes,
And perfects them at last; and in the depths
Of His divine forbearance, suffereth long,
And passeth by transgression. That vast throng,
The multitude of peoples, nations, tongues,
Shall stand before His Throne, and every act
Of human kindness He will own as His,
And crown, is service rendered unto Him.
—*Plumtre.*

**Reading.**

We live truly, exactly in proportion as we go out of ourselves and enter into the fulness of the experience of those whom we serve, and by whom in turn we are served.—*Westcott.*

The trivial services of social life are best performed, and the lesser particles of domestic happiness are most skilfully organised, by the deepest and fairest heart. It is an error to suppose that homely minds are the best administrators of small duties. Who does not know how wretched a contradiction such a rule receives in the economy of many a home? Where is the presiding genius of a home, taste and sympathy unite (and in their genuine forms they cannot be separated) —the intelligent feeling for moral beauty, and the deep heart of domestic love—with what ease, what mastery, what graceful disposition do the seeming trivialities of existence fall into order, and drop a blessing as they take their place! By the self-renunciation of affection there comes a spontaneous readjustment of various wills; every day has its silent achievements of wisdom, and every night its retrospect of piety and love—and the tranquil thoughts that in the evening meditation come down with the starlight seem like the Serenade of Angels, bringing in melody the Peace of God.—*Martineau.*

### Travel Notes.

By Lennox Carew.

### XXVIII.—THE COUNTRY ROUND SAN REMO.

### Excursions to the West and North of San Remo.

First, of course, comes Bordighera; this is so easy by train that it is hardly worth wasting the money for a carriage. The well-instructed reader will remember that Bordighera is closely associated with the patriot author, Ruffini, and that we first become acquainted with Doctor Antonio on the Bordighera road. It is now almost exclusively an English settlement, which makes it less interesting, and I should never settle there or at Ospedeletti when San Remo is so handy.

### Dolceacqua and Apricale.

Unless you have with you a decided invalid I should go to Bordighera by train and take a carriage from there; like almost all districts in this part of the world there are but few high roads, and one is forced to make long détours to reach places that are really close at hand, if only there was any road to them! If you go to Apricale, and it is a great pity to miss it, you will need a pair of horses. After a drive of some ten kilometres through delightful olive woods, and if in spring, over ground gemmed with wild flowers and blue flax, you come upon Dolceacqua, thrown into vivid relief against a background of snow mountains. The huge abandoned castle looks very impressive towering above the little town cowering about its feet, the Nervia flows under the bridge with its one strong mighty arch; the town itself is full of the usual stairways and arches, and is somewhat gloomy; all ways lead up to the mighty stronghold of the Dorias. Pursuing your way to Apricale you will pass Isolabonna. By this time you will be glad to stretch your legs and rest the horses again, and in a quarter of an hour you will have seen all the lions of Isolabonna. Another mile will bring you to Apricale. This place is dismissed by most guide books with scornful silence, but I assure you it is one of the most striking spots on the Riviera, the peculiarities of the fortress towns of the Littoral being accentuated here. The overhanging houses advance so much over yawning gulfs that they have to be supported on brackets and supports of various kinds. If any of your party are good walkers, you might return on foot to San Remo instead of touching at Bordighera; it is only about nine miles, and by so doing you would see some of the bye-ways and also San Romolo.

### Ventimiglia.

This can be easily done in a morning by train from San Remo, or by the diligence, or more luxuriously, by carriage. It is a singular old town and quite worthy a visit, though most people's acquaintance with it extends no further than getting through the Customs with what patience they can bring to bear on the subject. I delight in the old place, and artists and photographers (and in these days everyone is one or the other) will find themselves well placed.

### Excursions to the East and North.

Of lengthy ones, I think we must place Ceriana first. The drive costs from 14 francs, and must be made by the chapel of the Madonna de la Garde and Poggio. For the robust to walk is preferable; distance from San Remo six miles and a half. I did it in that way, starting very early in the morning, having the whole day to rest and sketch, and returning in the cool of the evening, and I did not find it at all too fatiguing. This part of the country suffered dreadfully in the earthquake of 1887. Ceriana felt it considerably, but nothing to Bussana, where, the earthquake happening on Ash Wednesday, several deaths occurred in the church, and the place was so ruined that it has never since been inhabited. At Bajardo the roof of the church fell in and buried the congregation, and the same tragedy occurred at Taggia, further along the coast.

### Taggia and Lampedusa.

You will not, I feel sure, be of my adventurous spirit and bravely locate yourself in Taggia itself, a lovely spot, but haunted by odours of all kinds, and by an absence of sanitation truly appalling; all the same the inhabitants are healthy and hardy, and seem to thrive on these varied smells. I feel that I could successfully pass an examination on the subject of Continental odours, their origin, growth, and effect, and I defy anyone to understand the matter more thoroughly. I was lodged in a corner of the Spinola Palace, which now calls itself an inn, and the charming landlord and his wife were all that was kind and delightful—it was not their fault that they were unaware that their ménage lacked anything; it was one of the most charming experiences of my nomad life. In the evenings the non-commissioned officers of the Bersaglieri detachment quartered there came in and danced and sang and talked to us in the most easy and delightful way, gracious, free, and courteous, and without a tinge of forwardness. Their superior officers generally dined with us and with the landlord, and we were like a happy family. All the same, I recommend the general public to study Taggia in a day's excursion. You enter the little town through a grim gateway and a very narrow street, only just wide enough for vehicles to pass; high above you on each side tower the houses, not too clean I must admit, but almost all

The Entrance to Taggia.

having a display of brilliant flowers. Those unable to walk must give up Lampedusa unless they can trust themselves to a mule. You ascend first to Castellaro, where the church, like that of Taggia, was destroyed in 1887. Looking back as you ascend towards Castellaro, you see the house of Signora Eleanora, mentioned in "Doctor Antonio." After seeing Castellaro, which in itself is not of interest, but to which the walk is exquisite, you turn to the left, and in course of time (about half an hour) arrive at Lampedusa, a poor enough place, but interesting from its legends and the magnificent views all round. There is a very singular bridge which crosses the valley at Taggia, consisting, if I remember rightly, of sixteen arches, with a shrine on it to commemorate the marvellous escape of two children in the earthquake of more than sixty years ago.

### TRAVEL NOTES AND QUERIES.

ARCACHON (Espérance).—The roads are very good for cycling in most directions. As for a small furnished house, I am doubtful; the general plan is to let flats. I think it might be difficult to get anything very reasonable. The climate would be suitable, I think, for your purpose, chiefly because there is so much sun; but it is not bracing, though the air is fresh and delightful. It is fairly lively, and suitable for an elderly person—nice walks and good boats. All information to be found in a Travel Article in THE HOSPITAL in February; I think it was headed "Bordeaux and Arcachon." I do not think any place in Germany quite so good for your purpose as Arcachon, though Heidelberg is delightful. Fare, first, return, £5 7s. 8d.; second, £3 15s. 2d. Other expenses about the same, probably a little less.

WESTERN SCOTLAND (Mayflower).—Am absent from town, and separated from papers, but I am quite sure that you will not find temperance hotels anywhere in Scotland, except in large towns. Callander is an excellent place for visiting the Trossachs, close to Loch Lomond. I think not so very expensive, but, alas, Scotland is always dear. I think you are planning too much. I should go through to Glasgow and Oban, stay there a week; go to Callander and visit the Trossachs; and home straight, viâ Edinburgh. Hotels are very expensive, and I think that is the limit you can manage. I do not think you will find anything so cheap as Gaze's coupons in Scotland. A week at Oban you might possibly manage cheaper, and then take a fortnight's tickets.

*(For Travel Advertisements see Page xii.)*

### Presentations.

NATIONAL HOSPITAL FOR THE PARALYSED AND EPILEPTIC.—A silver tea service, subscribed for by the resident medical and nursing staff and some former members, together with an address, has been presented to Miss Rachael F. Tweed upon her retirement. Dr. Collier, senior house physician, made the presentation, and expressed the deep regret felt at Miss Tweed's resignation. At the same time Miss Tweed received a present of plate from the hall porters and the other members (male and female) of the domestic staff. Among other tokens of goodwill and regard offered to Miss Tweed are a cheque from the Board of Management and personal gifts from her chief fellow workers.

BRENTFORD COTTAGE HOSPITAL.—Miss L. Stone, who has just been appointed Matron of the Woodford Jubilee Hospital, has, in recognition of her services as matron of Brentford Cottage Hospital, been presented with a handsome gold watch from the medical officer, Dr. Bolt. The townspeople have also presented her with a handsome Davenport as a mark of their esteem.

### Novelties for Nurses.

### MESSRS. HARRIS'S EXHIBITION OF ART NEEDLEWORK, 25, OLD BOND STREET, W.

IT is always a delight to nurses to have a piece of pretty needlework on hand. The work on linen with linen threads which is the principal feature of Messrs. Harris's display has many advantages. It is varied, effective, and beautiful, whilst a careful bath does it no harm. It would be impossible to enumerate the novelties of the season, but we may mention that appliqué designs have been carried out with great boldness and success. Portières, table centres, covers, cushions, chair seats and backs—a fashionable whim just now—veil rolls, waterproof cushion bags, as well as church embroidery, give a large range of choice to intending purchasers.

### Notes and Queries.

**The contents of the Editor's Letter-box have now reached such unwieldy proportions that it has become necessary to establish a hard and fast rule regarding Answers to Correspondents. In future, all questions requiring replies will continue to be answered in this column without any fee. If an answer is required by letter, a fee of half-a-crown must be enclosed with the note containing the enquiry. We are always pleased to help our numerous correspondents to the fullest extent, and we can trust them to sympathise in the overwhelming amount of writing which makes the new rules a necessity.**

**Every communication must be accompanied by the writer's name and address, otherwise it will receive no attention.**

*Ward Prayers.*

(117) Will you kindly tell me where I can get a book of prayers for ward use?—*L. Stone.*

There is no really good book of prayers for ward use. Some matrons prepare or adopt prayers for this purpose.

*Capetown.*

(118) Will you kindly tell me the name of a hospital or nursing institution in Capetown, South Africa, as I am desirous of obtaining a post out there?—*Nurse M.*

The New Somerset Hospital, Capetown (matron, Sister Alicia), is the largest hospital; and the Victoria Nurses' Institute, The Hoff, Capetown, was founded in memory of the Jubilee.

*Nurses' Clubs.*

(119) I should be so glad if you would tell me how I could join a nurses' club in London and the addresses of some. I am shortly joining a home there, and should like to belong to a club where one would be in touch with the nursing world.—*Clublands.*

The Royal British Nurses' Association possesses a nice club at 17, Old Cavendish Street, W., which is open to members of the Association. The Midwives' Institute and Trained Nurses' Club, at 12, Buckingham Street, Strand, admits trained nurses who are not midwives as associates.

*Three Years' Training.*

(120) Will you kindly tell me if you think any matron would take me for three years' training? I have had one year in a general hospital (30 beds). After that I did three years private nursing, and I hold a maternity certificate from the British Lying-in Hospital. I could have excellent references for my work. My age is 26, and I am tall, strong. If you can help me I shall be grateful to you.—*Lena.*

It is, of course, difficult to persuade matrons to accept probationers partially trained, but with your qualifications it ought not to be impossible. There is an excellent opportunity at the infirmary, Havil Street, Camberwell, just now. The training school has lately been organised, and the authorities have had some difficulty in getting the kind of nurse they want. Your best chance of acceptance lies with the Poor Law authorities.

*Provincial Nursing Associations.*

(121) Could you kindly give me the addresses of the Nurses' Co-operation in Liverpool, Manchester, and one or two more large towns, if possible, in the provinces, or tell me how I may obtain them?

You will find a list of nursing associations managed by a committee in "The Nursing Profession: How and Where to Train." The price is 2s., and the publishers the Scientific Press, W.C.

*Work Abroad.*

(122) Sister Edith would much like to know if there is any nursing co-operative association in Rome for the winter months; (2) and also how appointments to the Civil Government hospitals abroad are obtained. She has had four and a half years' hospital training in one of the large London hospitals, and has held the post of sister in a provincial one.

The Hollond Institute, 1, Tavistock Chambers, W., has a branch in Rome, and "An Old Resident of Rome," in the "Mirror" of May 27th, mentions Miss Watson's Home. 2. The appointments in the Government Civil hospitals are filled up in different ways. Sometimes the vacancies are advertised, and the best candidates elected; at others matrons of our larger hospitals are asked to select them from personal knowledge; at others the nursing associations supply them. The Colonial Nursing Association, Imperial Institute, W., has sent out so far a very fair proportion.

*Dandruff.*

(123) Will you kindly tell me the name of the preparation (sulphur in some form, I think) recommended in THE HOSPITAL about two years ago for dandruff? Also, I would be much obliged if you will tell me where I could get it.—*A Private Nurse.*

"Dandruff and Baldness," by Leslie Phillips, M.D., in THE HOSPITAL for July 31st, 1897, is the article to which you refer. We recommend you to read it, as it gives directions how to take care of the hair. The pomade mentioned is Behrendorf's Pomade Soufrée, made by Messrs. May, Roberts, and Co., 9-11, Clerkenwell Road, E.C.

*Invalid Chair.*

(124) Can any of your readers kindly tell me where I could hire a propelling chair (indoor) for a week or fortnight to see if it would be suitable before buying one for an invalid gentleman.—*Mitcham.*

Most manufacturers would be open to an arrangement of the kind you mention. For names and addresses see our advertisement columns.

*Human Skeleton.*

(125) How can I obtain a human skeleton for anatomical study? I cannot afford to pay much.—*Student.*

You might advertise in the medical journals or in the *Exchange and Mart*; but if you do not wish to keep it after your course of study is ended, would it not be cheaper to hire one? Make your want known to your local medical and surgical appliance supply agents.

## Figures and Tables

**Figure f1:**
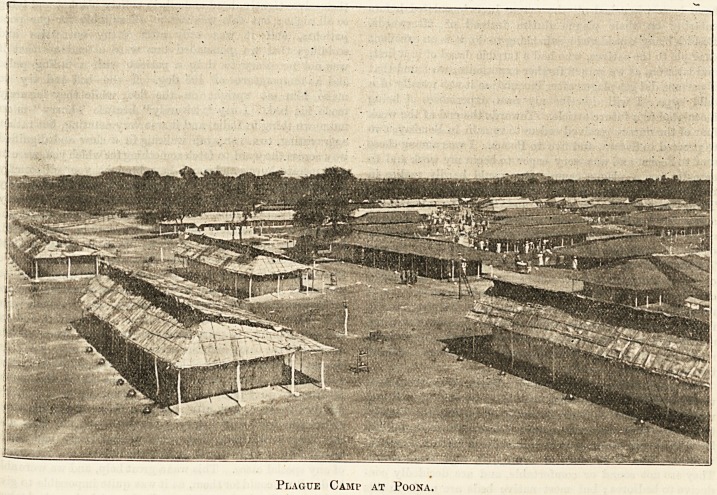


**Figure f2:**